# *In vitro* Antiviral Activity of *Rubia cordifolia* Aerial Part Extract against Rotavirus

**DOI:** 10.3389/fphar.2016.00308

**Published:** 2016-09-13

**Authors:** Yuanyuan Sun, Xuepeng Gong, Jia Y. Tan, Lifeng Kang, Dongyan Li, Vikash, Jihong Yang, Guang Du

**Affiliations:** ^1^Department of Pharmacy, Tongji Hospital, Tongji Medical College, Huazhong University of Science and TechnologyWuhan, China; ^2^Department of Pharmacy, National University of SingaporeSingapore, Singapore; ^3^Department of Infectious Diseases, Wuhan Union Hospital, Tongji Medical College, Huazhong University of Science and TechnologyWuhan, China; ^4^College of Life Sciences, Central China Normal UniversityWuhan, China

**Keywords:** *Rubia cordifolia*, aerial part, rotavirus, antiviral activity, apoptosis, diarrhea

## Abstract

The root of *Rubia cordifolia* has been used traditionally as a hemostatic agent, while the aerial part of the plant consisting of leaf and stem is known to exhibit anti-diarrheal properties and has been widely used as a remedy in many parts of China. As rotavirus is one of the most commonly associated diarrhea-causing pathogen, this study aims to investigate the anti-rotaviral effect of *R. cordifolia* aerial part (RCAP). The cytotoxicity of RCAP toward MA-104 cells was evaluated using the WST-8 assay. Colloidal gold method and real time polymerase chain reaction (qPCR) assay were used to confirm the findings of the antiviral assay. Then, 4′,6-diamidino-2-phenylindole (DAPI) staining method was subsequently used to investigate the mode of death among the cells. And the representative components of aqueous extract were isolated and identified. It was shown that both the viability of MA-104 cells and the viral load were reduced with increasing concentration of the extract. DAPI staining showed that virus-induced apoptosis was the cause of the low cell viability and viral load, an effect which was accelerated with incubation in the aqueous herbal extract. The major compounds postulated to exhibit this activity were isolated from the aqueous herbal extract and identified to be compounds Xanthopurpurin and Vanillic Acid. This study showed that RCAP extract effectively inhibited rotavirus multiplication by promoting virus-induced apoptosis in MA-104 cells.

## Introduction

Rotavirus, pathogenic to both man and animals, is a non-enveloped virus with a triple-layered double capsid and 11 segments of double-stranded Ribonucleic acid (RNA; Goncalves et al., 2005). It is the most common diarrhea-causing pathogen in children world-wide (Parashar et al., 1998; Cecilio et al., 2012). Every year, two million children are hospitalized, and 800,000 deaths occur due to rotavirus-associated diseases (Atherly et al., 2009; Mameli et al., 2012). The virus is transmitted via the oral-fecal route and the disease is a consequence of viral replication inside the small intestine (Davidson et al., 1975; Tallett et al., 1977). Clinical manifestations of rotavirus gastroenteritis are typically fever, vomiting, abdominal pain, diarrhea and dehydration (Walker-Smith, 1978; Cecilio et al., 2012; Paul et al., 2014).

Progress has been made in recent years in the discovery of anti-viral drugs such as chemicals, probiotics, immunoglobulins and natural products (Heaton and Ciarlet, 2007; Teran et al., 2009; Grandy et al., 2010; Knipping et al., 2012). Some commercially available chemical drugs have since been examined in clinical trials to have shown promising anti-rotaviral activities (Gu et al., 2000). Ribavirin was reported to exert its anti-rotavirus activity by inhibiting guanosine 5′-monophosphate biosynthesis (Schoub and Prozesky, 1977; Smee et al., 1982). Cimetidine and famotidine showed broad spectrum antiviral activities (Kabuta et al., 1989; Bourinbaiar and Fruhstorfer, 1996) and have been proven to be effective in rotavirus gastroenteritis (Li et al., 1995). Nitazoxanide was also reported to shorten the duration of diarrhea and hospitalization caused by rotavirus (Rossignol et al., 2006; Teran et al., 2009). However, their severe side effects were noted in clinical trials (Kim et al., 2012). Probiotics have also been explored to treat diarrhea caused by rotavirus. However, it remains unclear which type of probiotics are useful and the appropriate dose for children remained questionable (Kolader et al., 2013). In addition, high costs of probiotics ranging from US$10 to US$15 for a 5-day treatment deters them from being used in the treatment of rotavirus-induced diarrhea, especially in developing countries. Thus, the replacement of fluids and electrolytes remains the gold standard for the management of rotaviral gastroenteritis (Fischer Walker et al., 2011). Although fluid and electrolyte replacement may be beneficial to patients who are malnourished and with persistent diarrhea, this approach may not be fully effective in alleviating the rotavirus-induced diarrhea in children. The efficacy of an agent against the rotavirus and a good side effect profile are therefore essential considerations. To this end, antiviral agents from natural sources served as promising candidates for new drug discovery (Kwon et al., 2010; Chingwaru et al., 2011; Tam and Roner, 2011).

*Rubia cordifolia* (Qiancao in Chinese), also known as Madder, is a perennial climber that is widely distributed in China and India (Li et al., 2009; Do et al., 2013). In Traditional Chinese Medicine, the extract of *R. cordifolia* root has blood coagulating properties. Recently, several studies have reported a variety of medicinal properties including anti-tumor, anti-inflammatory, antimicrobial and apoptotic effects from its root (Zhou et al., 2012; Do et al., 2013; Kaur et al., 2014). The aerial part of *R. cordifolia*, referred to as *R. cordifolia* aerial part (RCAP), consisting of leaf and stem is widely used in Hanzhong city of Shaanxi Province, China, for the treatment of diarrhea in children. In light of this, we aim to investigate the biological mechanism underlying the anti-diarrheal effect of RCAP by studying their antiviral property, particularly the RCAP aqueous extract against rotavirus.

In this study, we first evaluated the cytotoxicity of the aqueous extract of RCAP on healthy MA-104 cell line. The extract was then incubated with virus-infected cells to assess its anti-viral efficacy. The effects of the rotavirus-induced apoptosis were studied in the presence of the extract. To our knowledge, this is the first study aimed to elucidate the anti-rotaviral activity of the RCAP.

## Materials and Methods

### Preparation of Extract

RCAP was collected from Hanzhong city of Shaanxi Province. All the plant materials were air dried, powdered and deposited at the Department of Pharmacy, Tongji Hospital. Hot water extract of the plant was prepared according to the procedures as described. The plant (1 kg) was boiled in 1 L of distilled water for 1 h. The aqueous solution was collected and the residual was extracted again with another 1 L of distilled water. This step was repeated, until the extract became transparent. All of the aqueous extracts were combined, filtered through gauze, concentrated under low pressure and lyophilized. The lyophilized powder was dissolved in sterile distilled water.

### Cell and Virus Culture

The rhesus monkey kidney cell line MA-104 and human rotavirus G9 were obtained from Dr. Jihong Yang. The MA-104 cells were cultured in Dulbecco’s modified Eagle’s medium (DMEM) supplemented with 10% v/v fetal bovine serum (FBS; Hyclone Laboratories Inc., USA) and 1% v/v of 10,000 unit/mL penicillin and 10,000 μg/mL streptomycin. The cell cultures were maintained at 37°C in a humidified 5% CO_2_ atmosphere under aseptic condition. Rotavirus was activated with 15 μg/mL trypsin for 30 min at 37°C and propagated in MA-104 cells monolayer in the presence of 5 μg/mL trypsin. The virus titer was estimated from cytopathogenicity by limit-dilution method and expressed as 50% tissue culture infectious dose (TCID_50_/mL) by the Reed and Muench (1938) statistical method.

### Determination of the Maximum Non-toxic Concentration

The cytotoxicity of the tested extract was evaluated using WST-8 assay (Tominaga et al., 1999). MA-104 cells (5000 cells/well) were grown in 96-well plate for 24 h. Different concentrations of aqueous extract were applied to culture wells in quadruplicate (1.95, 3.91, 7.81, 15.63, 31.25, 62.5, 125, 250, 500, 1000 mg/mL). After incubation at 37°C, 5% CO_2_ for 48 h, 10 μL of WST-8 solution was added to each well. Plates were then incubated at 37°C for 2 h. A microplate reader was used to test the absorbance of each well at 450 nm. The control for this assay was monolayer MA-104 cells incubated only with DMEM. Results were estimated by regression analysis. Cell viability was calculated as OD_ex_ / OD_con_ × 100%. OD_ex_ indicates the absorbance of extract treated group. OD_con_ indicates the absorbance of the control cells without the extract. The highest concentration of the extract which showed no significant statistical difference from the controls in cell viability was deemed as the maximum non-toxic concentration (MNTC).

### Antiviral Assay

Antiviral assays used in this study have been described by Barnard et al. (1997). In the mixed treatment assay: Extract diluted with DMEM containing 5 μg/mL trypsin was mixed with rotavirus at various concentrations (0.12, 0.24, 0.49, 0.98, 1.95, 3.91, 7.81, 15.63, 31.25, 62.5 mg/mL), which were then incubated at 37°C for 2 h. The mixtures were inoculated in quadruplicate onto near confluent MA-104 cells monolayer in two 96-well plates, each well containing 100TCID50 of the rotavirus. The cells were incubated at 37°C with 5% CO_2_ atmosphere for 48 h and observed with an inverted microscope. Then, WST-8 assay was done in one 96-well plate as previously described and the supernatant of each group in the other plate was gathered and tested with the rotavirus detection kit (Wantai BioPharm, Beijing, China) at 4°C. Cell viability was calculated as OD_rv_ / OD_con_ × 100%. OD_rv_ indicated the absorbance of rotavirus infected group with or without RCAP extract. OD_con_ indicates the absorbance of non-infected cells without RCAP extract (control).

In the post treatment assay: 100TCID_50_ of the rotavirus was inoculated onto the near confluent MA-104 cells monolayer for 1 h. Extract of various concentrations (0.12, 0.24, 0.49, 0.98, 1.95, 3.91, 7.81, 15.63, 31.25, 62.5 mg/mL) diluted with DMEM containing 5 μg/mL trypsin were added in quadruplicate. The cell viability was tested and the content of each group was gathered as described before.

### RNA Extraction

The contents gathered from the antiviral assay were used in the extraction of the rotavirus RNA. RNAs were extracted by a TRI Reagent method (Kim et al., 2014). For TRI Reagent extraction, 500 μL of each sample was incubated with 500 μL TRI reagent (Sigma-Aldrich Chemical Company) for 10 min at room temperature, followed by supplementation with 100 μL chloroform. After vigorous mixing for 10 s and centrifugation at 13000 rpm for 15 min at 4°C, the upper layer was transferred into a fresh tube. An aliquot of 400 μL iso-propanol was added and the resulting mixture incubated for 10 min at room temperature, followed by centrifugation at 13000 rpm for 10 min. After centrifugation the supernatant was discarded, and the remaining was washed with 1 mL 75% ethanol and air-dried for 10 min. RNA was dissolved in 20 μL RNase-free water.

### Quantification of Rotavirus by Real Time Polymerase Chain Reaction (qPCR) Assay

(i) Complementary DNA (cDNA) synthesis – the cDNA synthesis was conducted in a 25 μL reaction container using M-MLV Reverse Transcriptase. Firstly, 4 μL of extracted viral RNA, 1 μL of each primer and 11 μL RNase-free water, were incubated at 70°C for 10 min and then quickly chilled on ice for 5 min. After this, 5 × reaction buffer was added, 1 μL of each deoxynucleotide (dNTP), 1 μL RNasin Ribonuclease inhibitor and 1 μL M-MLV Reverse Transcriptase. The reaction was incubated for 60 min at 37°C, 5 min at 70°C. The cDNAs were maintained at 80°C until required. (ii) Primers – the primers used for rotavirus protein (VP6) region amplification were: Rota A (forward primer): 5′-GACGGVGCRACTACATGGT-3′ (V = A/C R = A/G) and Rota A (reverse primer): 5′-GTCCAATTCATNCCTGGTGG-3′ (N = A/G), which amplify a 379-bp product (Kang et al., 2004). They were synthesized commercially (Takara Bio Inc., China). (iii) The qPCR assay was performed on a CFX96 Touch^TM^ Real-Time PCR Detection System (Bio-Rad, USA) using SYBR premix Ex Taq. The assay was carried out in a total volume of 25 μL reaction mixture containing 12.5 μL SYBR premix Ex Taq master mix, 2 μL of cDNA, 1 μL of each primer and 8.5 μL RNase-free water. The optimized cycling conditions were as follow: initial denaturation at 95°C for 10 s, followed by 40 cycles of denaturation at 95°C for 10 s, primer annealing and extension at 56°C for 30 s. Fluorescence signals were recorded at the end of each cycle. A melt curve analysis was measured following amplification to confirm the specificity of the amplified products. Melting curve analysis consisted of 65°C for 5 s, and followed by increase in temperature to 95°C for 5 s with continuous fluorescence reading. RNase-free water, 5 × reaction buffer, dNTP, RNasin Ribonuclease inhibitor, SYBR premix Ex Taq used in qPCR were all obtained from Takara (Takara Bio Inc., China).

### 4′,6-diamidino-2-phenylindole (DAPI) Staining Assay

Cell nuclear morphology was evaluated by fluorescence microscopy following DAPI (Roche Diagnostics GmbH., Germany) staining. After gathering the contents from the antiviral assay, the monolayer consisting of MA-104 cells was washed once with DAPI-methanol (working solution, 1 μg/mL). Then, the cells were covered with DAPI-methanol and incubated for 15 min at 37°C. The staining solution was discarded after 15 min of incubation and the sample was washed once with methanol. The 96-well plates were examined under a fluorescence microscope (Nikon Eclipse T*i*, Nikon Instruments Inc., USA). Different concentrations of aqueous extract were added to MA-104 cells for 48 h in another 96-well plate without incubation with rotavirus. The cells were observed under an inverted microscope. Then, DAPI staining was performed after discarding the supernatant to observe the effects of the extract on the cells, before and after treatment with RCAP extract.

### Isolation and Identification of the Representative Components

The aqueous extract of RCAP was partitioned with ethyl acetate, CHCl_3_ and n-butanol, consecutively. Comparing the volumes of each fraction, ethyl acetate fraction was chromatographed on silica gel column (Qing-dao Marine Chemical Inc., China). The column was washed and eluted, then the eluates were separated and confirmed by thin-layer chromatography (Yantai Chemical Industry Research Institute, China). For further purification of active components, semi-preparative HPLC was carried out on a Dionex quaternary system with a diode array detector at a flow rate of 2.5 mL/min using a reversed-phased C18 column (5 μm, 10 × 250 mm, YMC-pack ODS-A). The representative active compounds isolated were identified by analyzing them on a Bruker AM-400 spectrometer (Bruker Corporation, USA).

### Statistical Analysis

Study results were analyzed using SPSS software (ver. 18.0; SPSS, Inc., USA). Results were presented as mean ± standard deviation (SD). One-Way ANOVA test was used to calculate differences. Statistical significance was indicated at *p* < 0.05. Curves of real-time qPCR were analyzed using CFX Manager Software (ver. 3.0; Bio-Rad, Inc., USA).

## Results

### Cytotoxicity of the RCAP Extract in MA-104 Cells

**FIGURE 1:**
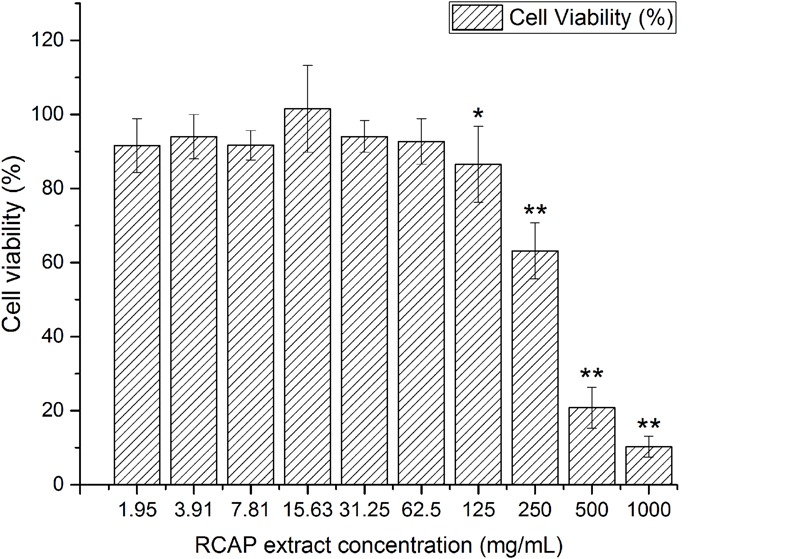
**Cytotoxicity of RCAP extract in MA-104 cells.** The non-toxic groups were determined by comparing the cell viability. Cell viability was calculated as OD_ex_ / OD_con_ × 100%, and OD_con_ = 1.74 ± 0.06. Data are represented as mean ± S.D. *n* = 11. ^∗^*p* < 0.05 vs mock; ^∗∗^*p* < 0.01 vs mock by one-way ANOVA test, *post hoc* Tukey test.

It was shown that there was no significant difference between the control and the extract treated group in the aspect of cell viability, for RCAP concentrations up to 62.5 mg/mL (**Figure 1**). Based on this result, the concentrations of extract that exhibited cytotoxicity were excluded from the subsequent antiviral assay and 10 concentrations (0.12, 0.24, 0.49, 0.98, 1.95, 3.91, 7.81, 15.63, 31.25, 62.5 mg/mL) were used.

### Antiviral Activity of RCAP Extract

**FIGURE 2:**
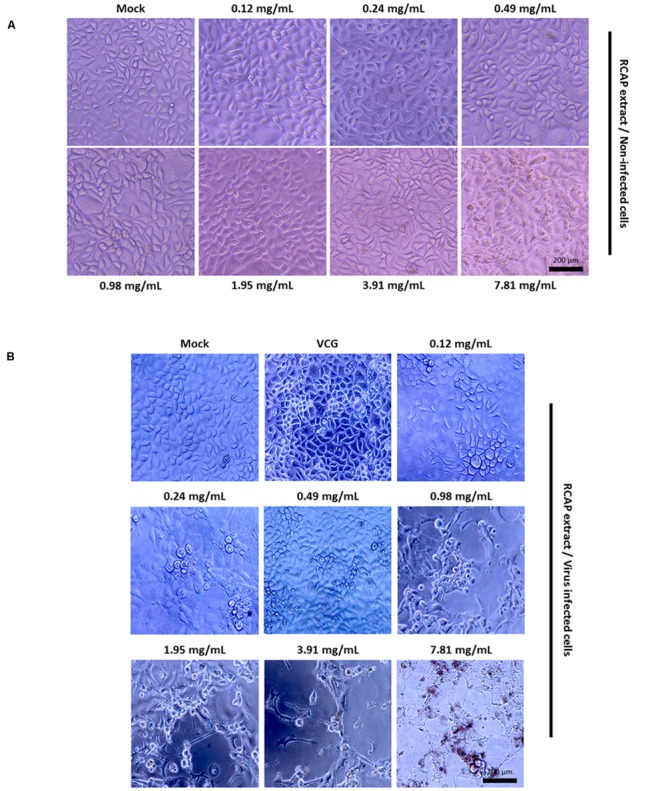
**Morphological observation of MA-104 cells in antiviral assay. (A)** Non-infected cells incubated with RCAP extract; **(B)** Rotavirus-infected cells treated with RCAP extract. Morphological changes were characterized by rounding and enlargement of the cells and by detachment of the cells from the substrate.

**FIGURE 3:**
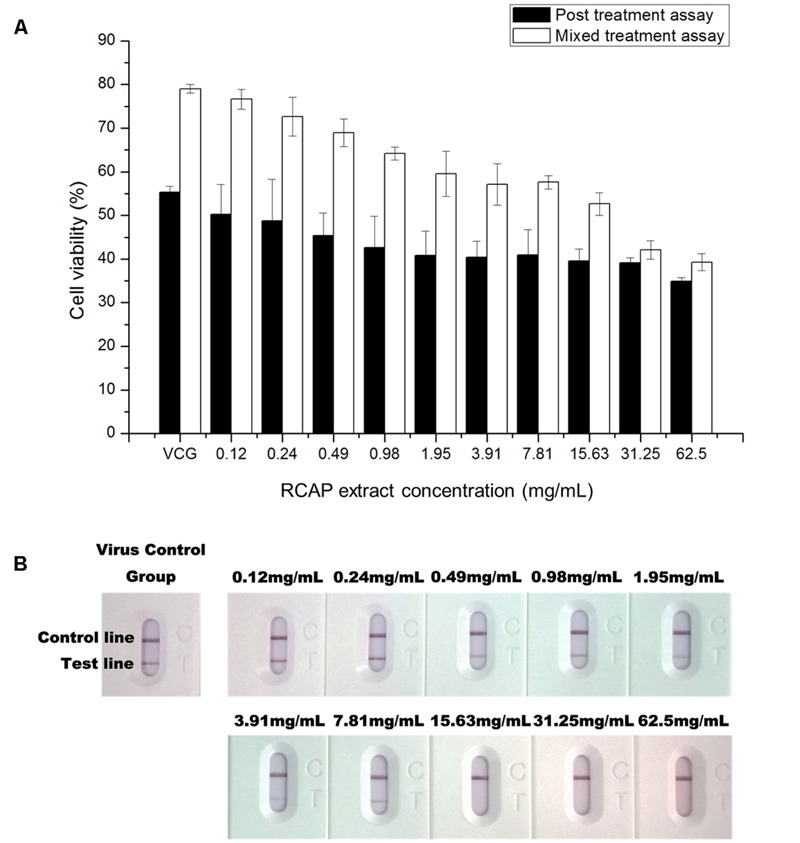
**Effect of RCAP on cell viability and viral load in antiviral assay. (A)** Cell viability of each group in mixed and post treatment assay. Cell viability was calculated as OD_rv_ / OD_con_ × 100%, OD_con_ of mixed treatment assay was 1.11 ± 0.03, OD_con_ of post treatment assay was 1.18 ± 0.03; **(B)** Detection of rotavirus in each group using the colloidal gold method. Control line indicates valid testing; test line indicates presence of rotaviruses.

In the mixed treatment assay, after various concentrations of extract and rotavirus were mixed and incubated, the mixtures were inoculated onto the confluent monolayer cells. The morphological changes of the virus infected or non-infected RCAP extract treated MA-104 cells were observed with light microscopy, after the cells in the virus control group (VCG, cells were only inoculated with virus) showed viral cytopathic effect (CPE). It was found that these affected cells increased with an increasing concentration of the extract (**Figure 2B**), while the morphology of non-infected cells treated with RCAP extract remained consistent regardless of the RCAP extract concentration (**Figure 2A**). This finding was consistent with the cell viability assay (**Figure 3A**). In the post-treatment assay, a similar situation was observed. As the extract concentration increased, more affected cells can be observed. There was indeed a negative relationship between cell viability and RCAP extract concentration (**Figure 3A**).

To study the virus quantitatively, a rotavirus detection kit was used on the supernatant isolated from each group. The color of the test line, indicative of the viral load, showed a reduction in intensity with increasing concentration of the extract. The color of the test line became undetectable when the extract reached a concentration of 15.63 mg/mL (**Figure 3B**). These results showed that the amounts of rotavirus decreased with increasing concentration of extract and could not be detected at higher concentrations of extract.

**FIGURE 4:**
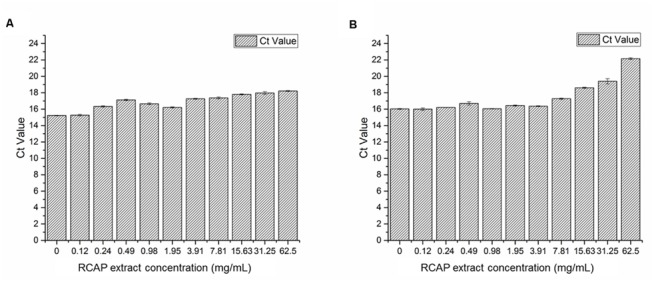
**Quantification of rotavirus by real time qPCR assay **(A)** Viral load in each group of mixed treatment assay; **(B)** Viral load in each group of post treatment assay.** *C*t values can be used as a quantitative measurement of the input target number which reflects the viral load in antiviral assay, and there was a negative relationship between *C*t value and the viral load.

To study the virus qualitatively, the SYBR Green-based qPCR assay was used. The contents (virus, the extract and cellular debris) collected from 10 different tested groups in mix treatment assay were examined. After cDNAs were prepared, the samples were quantified with ultraviolet spectroscopy. *C*t values can be used as a quantitative measurement of the input target number which reflects the viral load in antiviral assay, and there was a negative relationship between *C*t value and the viral load. The quantities of rotavirus were compared across treated groups by using their *C*t values. It was found the viral load was reduced as the concentration of the extract increased (**Figure 4A**). A similar trend was observed in post treatment assay as well (**Figure 4B**).

These results indicated that the extract exerted potential anti-rotavirus effect, not by affecting the rotavirus directly, but by acting on the infected MA-104 cells. The order of adding the rotavirus to the cells did not undermine the anti-viral effect of the extract. The antiviral effect was more pronounced when the virus was added to the cells prior to adding the extract, i.e., the post treatment assay.

### Effect of RCAP Extract on Infected MA-104 Cells

**FIGURE 5:**
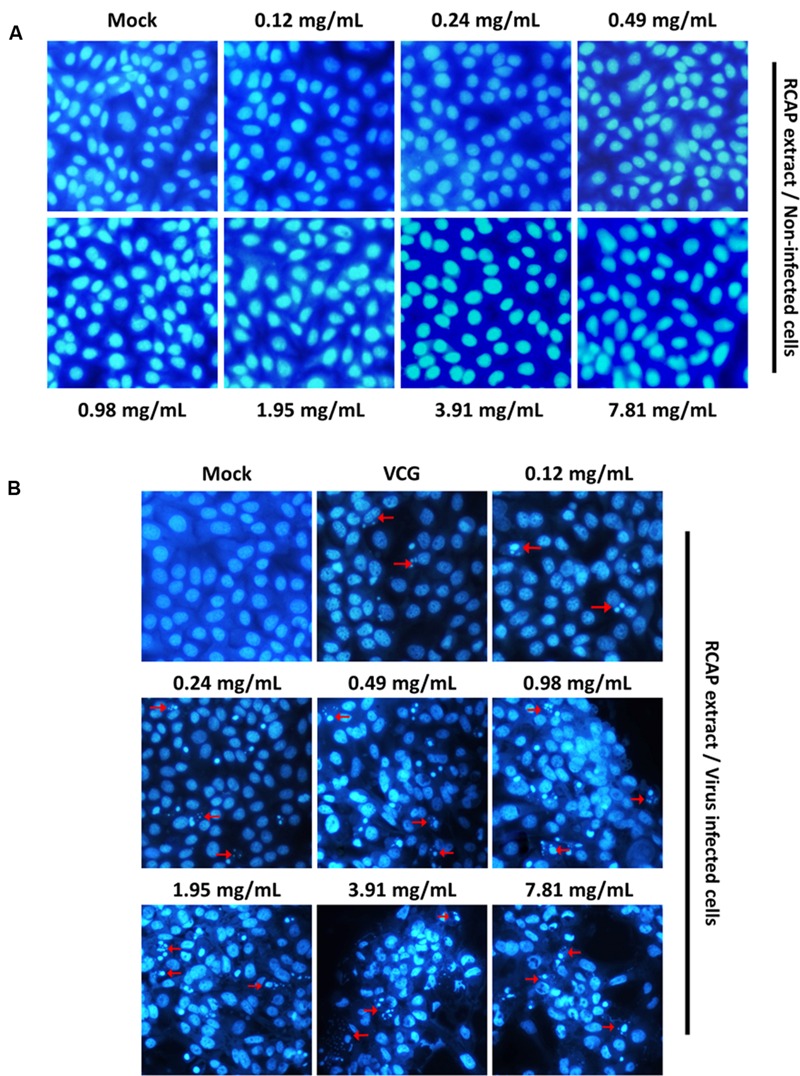
**Characteristic nuclear fluorescence of MA-104 cells with DAPI staining. (A)** Non-infected cells being treated with different concentrations of RCAP extract; **(B)** Rotavirus-infected cells being treated with different concentrations of RCAP extract. Red arrows correspond to nuclear fragmentation of apoptotic cells.

In our study, both the cell and viral viability decreased with increasing concentrations of the herbal extract. This may suggest that the death of MA-104 cells was not caused by the viral infection, as the amounts of viruses did not increase significantly with decreasing cell viability (Ward et al., 1984). Programmed cell death, also known as apoptosis, is defined as a physiological cell suicide process alternative to necrosis. Apoptosis can be differentiated from necrosis by their characteristic nuclear fluorescence when excited under fluorescence microscope. In our study, DAPI staining revealed apoptosis in MA-104 cells caused by rotavirus. The apoptosis was shown to be accelerated by the RCAP extract. The morphological changes associated with apoptosis such as chromatin condensation, nuclear fragmentation, and margination of nucleus were evident in MA-104 cells. Moreover, it was shown that the amounts of apoptotic cells increased with increasing concentrations of the extract (**Figure 5B**). The cells in the viral control group exhibited signs of apoptosis induced by rotavirus, though not as significant compared to the treatment group. However, the extract itself did not result in any apoptosis in the cells (**Figure 5A**). The results indicated that the elimination of rotavirus may be due to the apoptosis of virus-infected MA-104 cells accelerated by the herbal extract.

### Measurement of the Representative Components in RCAP Extract

**FIGURE 6:**
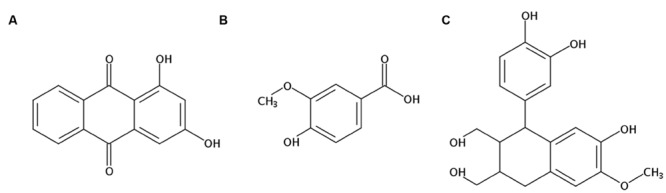
**Structures of components isolated and identified from the ethyl acetate fraction of RCAP aqueous extraction. (A)** 1,3-dihydroxy-9, 10-anthracenedione (xanthopurpurin); **(B)** 4-hydroxy-3-methoxybenzoic acid (vanillic acid); **(C)** 1-(3,4-dihydroxyphenyl)-1,2,3,4-tetrahydro-7-hydroxy-6-methoxy-2, 3-naphthalenedimethanol (isotaxiresinol).

There were three compounds isolated and identified from the ethyl acetate fraction of RCAP aqueous extract, which were 1,3-dihydroxy-9,10-anthracenedione (xanthopurpurin; **Figure 6A**), 4-hydroxy-3-methoxybenzoic acid (vanillic acid; **Figure 6B**) and 1-(3,4-dihydroxyphenyl)-1,2,3,4-tetrahydro-7-hydroxy-6-methoxy-2,3-naphthalenedi methanol (isotaxiresinol; **Figure 6C**). The extraction and separation processes, the NMR spectrums of these three compounds were shown in the Supplementary Material. Among them, xanthopurpurin and vanillic acid were considered to be responsible for the anti-viral effect observed with the RCAP aqueous extract.

## Discussion and Conclusion

*R. cordifolia* is widely used in China owing to the hemostatic effect of its roots (Zhang et al., 2005). Moreover, the water decoction of RCAP and a commercial medicine, namely, Erxieting^®^, obtained from RCAP, have long been used in clinical practice to treat diarrhea in children (Cui et al., 2005). Rotaviruses are the single most important cause of severe diarrhea (Fields et al., 2007). Therefore, we hypothesized that the anti-diarrheal effect of RCAP was to inhibit the replication of rotavirus. Based on the clinical applications of RCAP, we further hypothesized that the effective constituent exists in the aqueous extract of RCAP. Based on the hypotheses, we adopted the water decocting method to obtain the aqueous extract and studied its activity against rotavirus.

It was observed, for concentrations below 62.5 mg/mL, RCAP extract by itself did not exhibit any cytotoxicity. However, the cell viability decreased with increased extract concentration when the viruses were present. Initially, it was postulated that RCAP acted on rotavirus directly and promoted its proliferation because increased amounts of virus can reduce host cell viability. However, this was not the case observed in this study as the viral load did not increase with increasing extract concentration. This result motivated us to investigate further the mechanism of how RCAP extract induced cell death in rotaviral-infected cells.

Apoptosis is an active mode of cell death exhibiting a series of morphological and biochemical changes (Koyama et al., 1998; Galluzzi et al., 2008). Apoptosis can be induced by extrinsic signals, such as cytokines, or intrinsic stimuli that impinge on the integrity of mitochondria (Upton and Chan, 2014). Many animal viruses are known to induce apoptosis in infected cells, such as HIV, HBV and rotavirus (Basanez and Zimmerberg, 2001; Zhang et al., 2005).

Rotavirus has been reported to induce apoptosis in polarized epithelial cell lines, such as Caco-2 cells and HT-29 cells (Superti et al., 1996; Chaibi et al., 2005). In our study, we also observed apoptosis in MA-104 cells when incubated with 100TCID_50_ of the rotavirus for 48 h (**Figure 5B**). The results showed that not all the cells exhibited the characteristics of apoptosis. This is probably due to the heterogeneity in the stages of virus multiplication in each cell and that the rotavirus-induced apoptosis is time-dependent (Chaibi et al., 2005). However, when the extract was added, the amounts of apoptotic cells increased dramatically.

Although studies of apoptosis on *in vitro* viral replication revealed that host cell apoptotic responses can provide protection against viral infection (Everett and McFadden, 1999), rotavirus induced apoptosis by itself does not have an essential role in the host defense mechanism against viral infections. The multiplication of viruses may be slightly suppressed in the apoptotic cells, but that apoptosis in virus-infected cells cannot bring about the abortion of progeny virus formation. Rotavirus can grow significantly in cells undergoing apoptosis and is able to escape this host defense by completing multiplication before the onset of apoptosis. However, if the apoptotic process can be accelerated, then it can become a significant defensing mechanism against viruses.

In our study, we showed that below 62.5 mg/mL, the extract itself did not induce apoptosis (**Figure 5A**). On the other hand, the extract, which was administered together with viruses, accelerated apoptosis in a dose-dependent manner. From these results we concluded that, the extract may potentially accelerate the induction of rotavirus-induced apoptosis in MA-104 cells, with concomitant reduction of progeny virus production. The subsequent premature lysis by apoptosis of the rotavirus-infected cells can have a significant effect on the suppression of virus multiplication.

Considering the ethyl acetate fraction of RCAP water extraction had the largest volume of fractions with different polarity, we isolated and identified the representative components from this fraction. Xanthopurpurin is one of 10 dihydroxyanthraquinone isomers. Its molecular structure is similar to other anthraquinones occuring in madder, such as alizarin and purpurin (Derksen et al., 2002). Along with alizarin, purpurin, and other anthraquinones like mollugin, these major phyto-constituents reported in this plant have the function of activating caspase-3, a typical marker for cell undergoing apoptosis (Kim et al., 2009; Fotia et al., 2012; Tiwari et al., 2012; Tsang et al., 2013). Vanillic acid can induce apoptosis in HT-29 cell (Ho et al., 2009), and was deemed effective in the regulation of chronic intestinal inflammation by reducing the plasma level of interleukin 6 (IL-6; Kim et al., 2010). To control the immune response caused by pro-inflammatory cytokines (such as IL-1 and IL-6) during rotaviral infections is one of the major research directions of treating virus gastroenteritis (Azevedo et al., 2006). Thus, xanthopurpurin and vanillic acid may be regarded as potential anti-rotaviral reagents in the aqueous extract of RCAP, which requires further study.

This study has therefore demonstrated that the promotion of rotavirus-induced apoptosis is the main contributing mechanism of RCAP aqueous extract’s anti-viral property against rotavirus.

## Author Contributions

YS conducted all the experiments and composed this manuscript. XG and DL helped the experiments. JT and LK helped conducted the experiments and revised the manuscript. Vikash helped editing the manuscript. JY directed and helped to advise on the viral experiments. GD directed the entire study.

## Conflict of Interest Statement

The authors declare that the research was conducted in the absence of any commercial or financial relationships that could be construed as a potential conflict of interest.
