# Supplementary material for: In vitro Antiviral Activity of Rubia cordifolia Aerial Part Extract against Rotavirus
Source: Front Pharmacol. 2016 Sep 13;7:308. doi: 10.3389/fphar.2016.00308 (PMC5020101; doi:10.3389/fphar.2016.00308)
Supplement: Supplementary file 1 [file Data_Sheet_1.DOCX]

**Supplementary data**


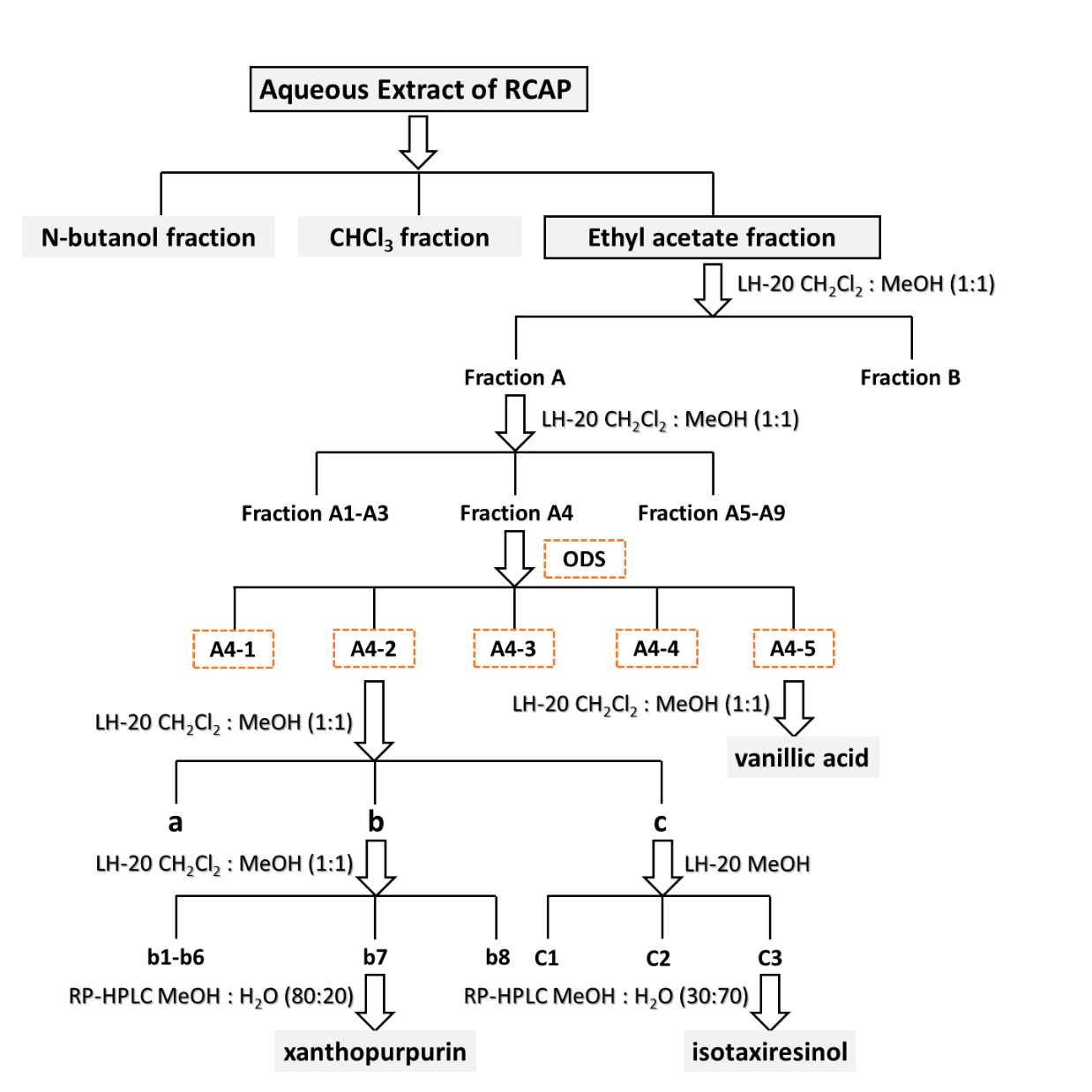
 Three compounds were isolated and identified from the ethyl acetate fraction of RCAP aqueous extract, which were 3-dihydroxy-9,10-anthracenedione (xanthopurpurin), 4-hydroxy-3-methoxybenzoic acid (vanillic acid) and 1-(3,4-dihydroxyphenyl)-1,2,3,4-tetrahydro-7-hydroxy-6-methoxy-2,3-naphthalenedimethanol (isotaxiresinol). The extraction and separation processes were shown in supplementary figure 1. Then the identification reflected by ^13^C-NMR spectrums of these compounds were shown in supplementary figure 2, supplementary figure 3 and supplementary figure 4, respectively.

Supplementary figure 1. Flowchart of the extraction and isolation processes of ethyl acetate fraction from RCAP aqueous extract.


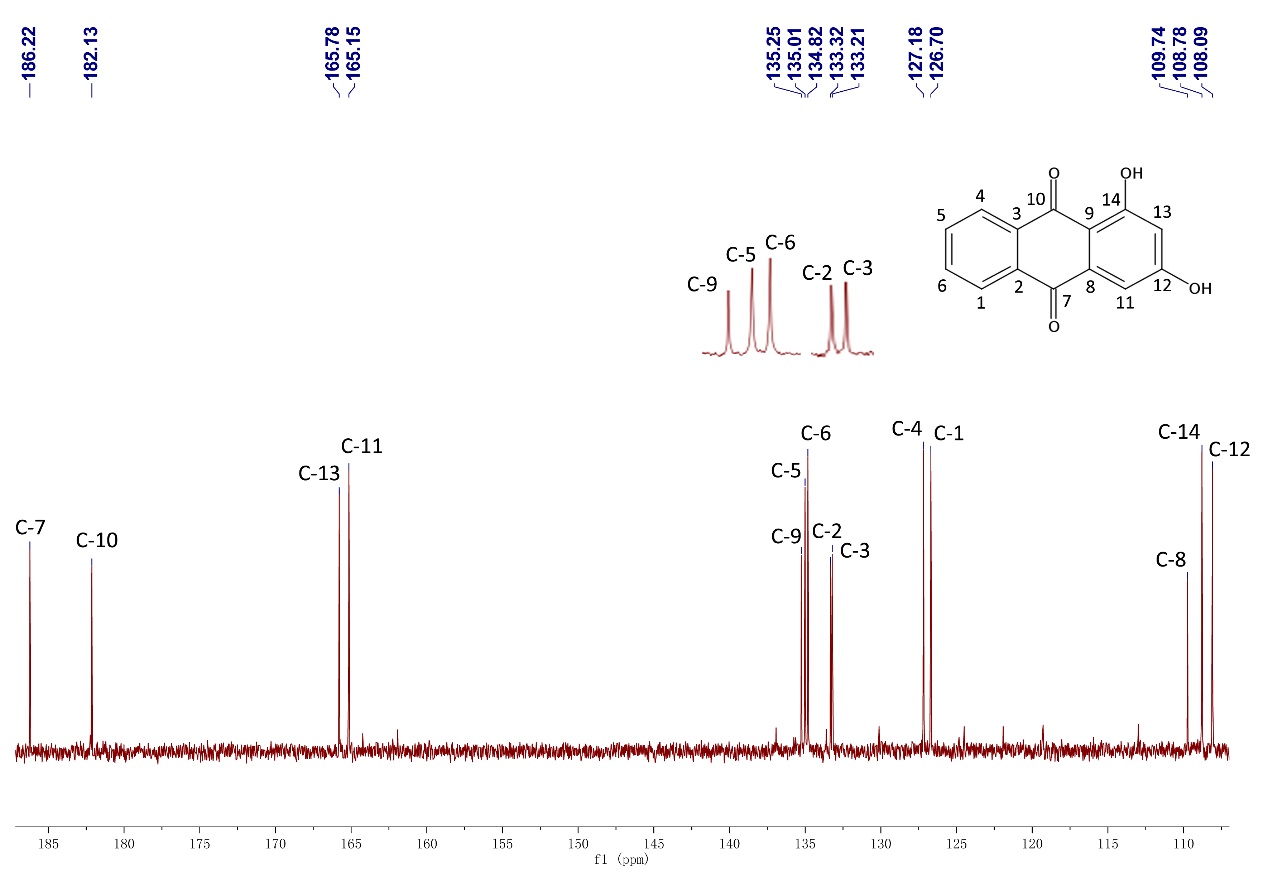
Supplementary figure 2. The ^13^C-NMR spectrum of 3-dihydroxy-9,10-anthracenedione.

3-dihydroxy-9,10-anthracenedione: ^1^H NMR (400 MHz, DMSO-d6), ppm, δ 5.73 (s, 1H), 6.59 (s, 1H), 7.72 (t, 1H, J=7.5), 7.83 (t, 1H, J=7.5), 8.07 (d, 1H, J=7.5), 8.13 (d, 1H, J=8.0)

^13^C-NMR (100 MHz, DMSO-d6), ppm, 108.09 (C-12), 108.78 (C-14), 109.74 (C-8), 126.70 (C-1), 127.18 (C-4), 133.21 (C-3), 133.32 (C-2), 134.82 (C-6), 135.01 (C-5), 135.25 (C-9), 165.15 (C-11), 165.78 (C-13), 182.13 (C-10), 186.22 (C-7).

Supplementary figure 3. The ^13^C-NMR spectrum of 4-hydroxy-3-methoxybenzoic acid.
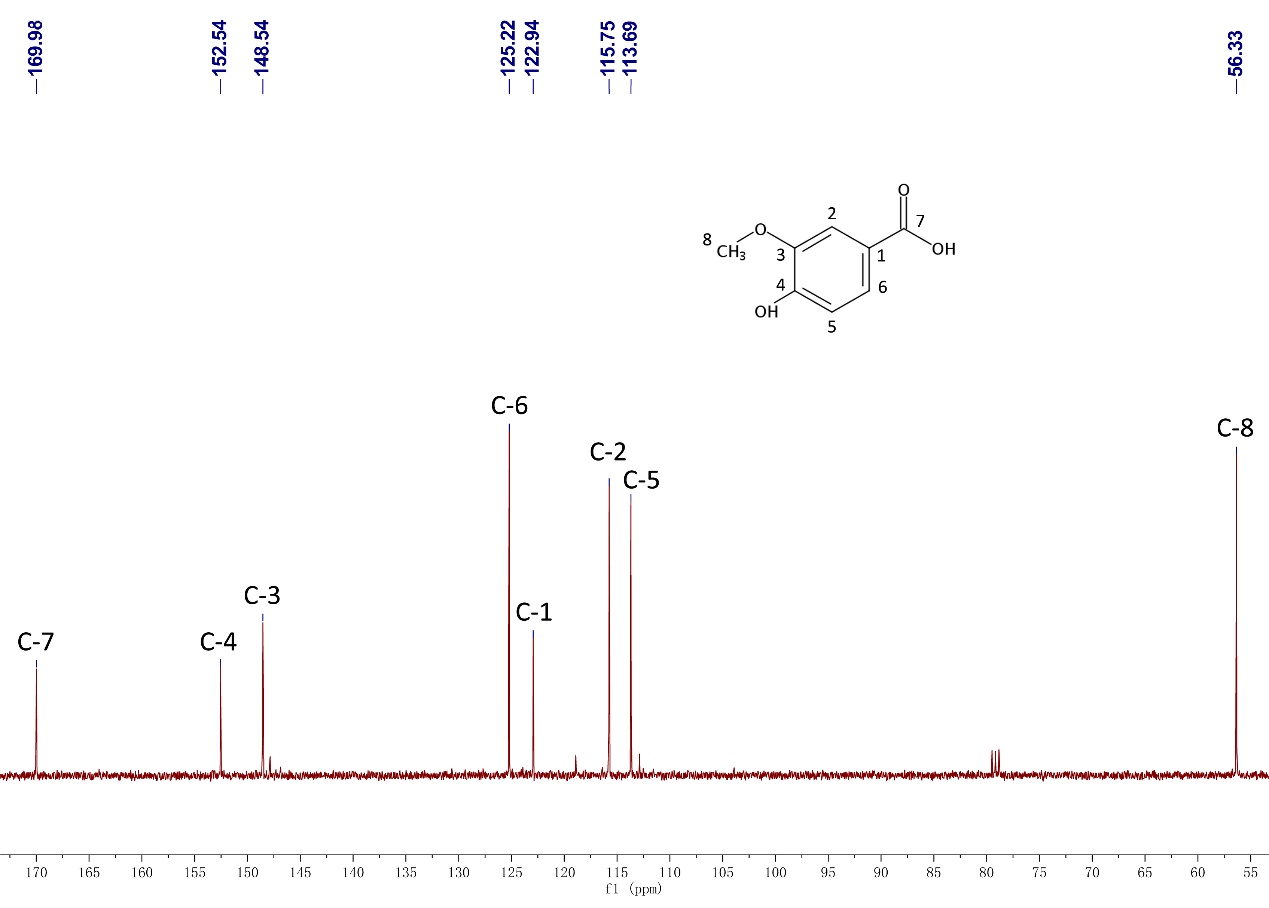


4-hydroxy-3-methoxybenzoic acid: ^1^H NMR (400 MHz, Methanol-d4), ppm, δ 7.57 (d, J = 1.9 Hz, 1H), 7.55 (d, J = 1.7 Hz, 3H), 6.84 (d, J = 1.9 Hz, 1H), 6.83 (d, J = 1.9 Hz, 1H), 3.88 (s, 3H, OCH3)

^13^C-NMR (100 MHz, Methanol-d4), ppm, 56.33 (C-8, O-CH_3_), 113.69 (C-5), 115.75 (C-2), 122.94 (C-1). 125.22 (C-6). 148.54 (C-3), 152.54 (C-4), 169.98 (C-7, -COOH)


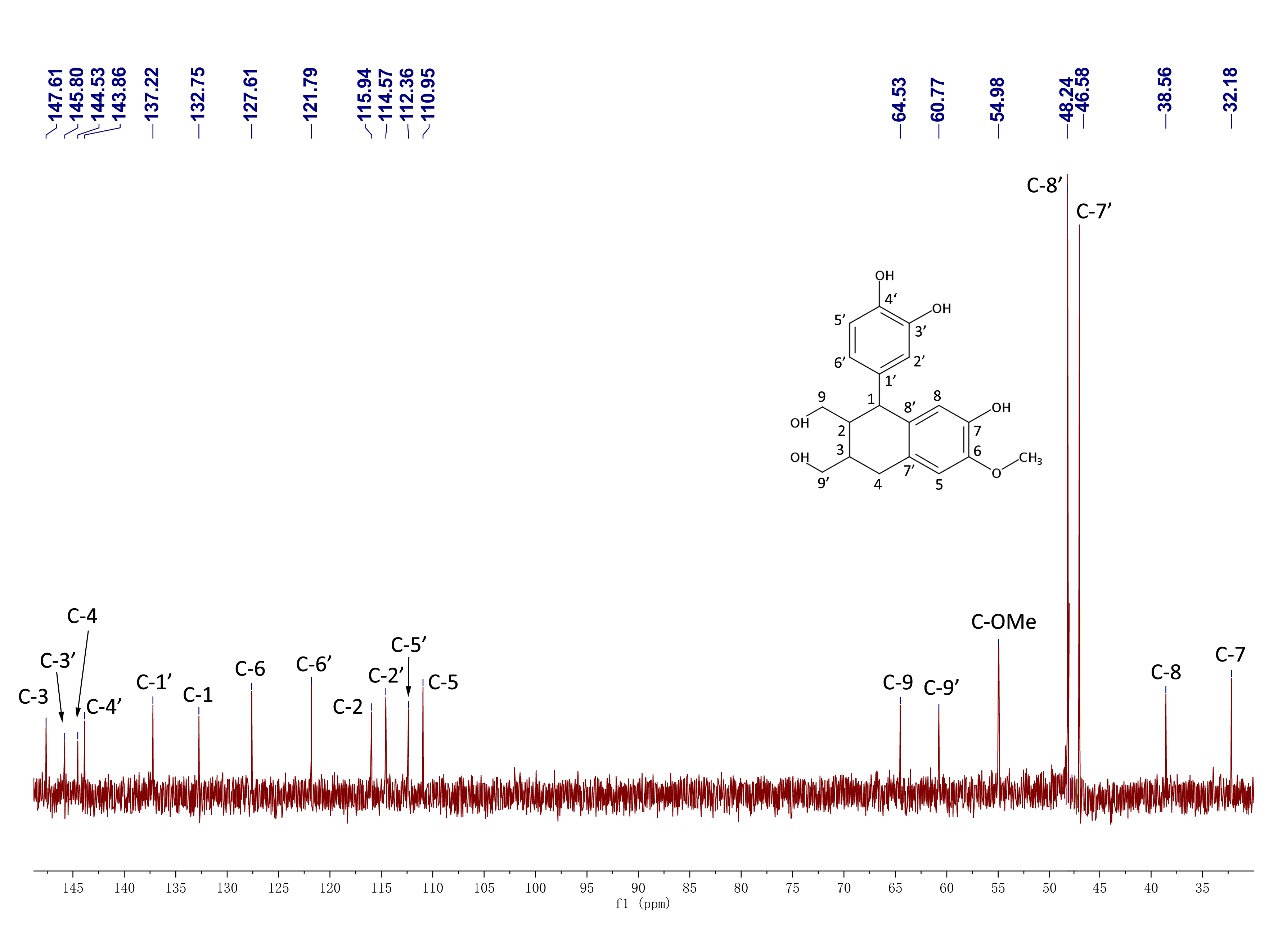


Supplementary figure 4. The ^13^C-NMR spectrum of 1-(3,4-dihydroxyphenyl)-1,2,3,4-tetrahydro-7-hydroxy-6-methoxy-2,3-naphthalenedimethanol.

1-(3,4-dihydroxyphenyl)-1,2,3,4-tetrahydro-7-hydroxy-6-methoxy-2,3-naphthalenedimethanol: ^1^H NMR (400 MHz, CD_3_OD), ppm, δ 6.69 (1H, d, J=8.0 Hz, H-5′), 6.61 (1H, s, H-5), 6.52 (1H, d, J=2.0 Hz, H-2′), 6.50 (1H, dd, J=2.0, 8.0 Hz, H-6′), 6.19 (1H, s, H-2), 4.67 (2H, m, H-9), 4.67 (1H, m, H-9′), 4.66 (1H, d, J=6.9 Hz, H-7′), 3.77 (3H, s, H—OMe), 3.40 (1H, dd, J=4.3, 11.1 Hz, H-9′), 2.73 (1H, br d, J=6.8 Hz, H-7), 1.97 (1H, m, H-8), 1.71 (1H, m, H-8′)

^13^C NMR (100MHz, CD_3_OD), ppm, δ 147.61 (C-3), 145.80 (C-3′), 144.53 (C-4), 143.86 (C-4′), 137.22 (C-1′), 132.75 (C-1), 127.61 (C-6), 121.79 (C-6′), 115.94 (C-2), 114.57 (C-2′), 112.36 (C-5′), 110.95 (C-5), 64.53(C-9), 60.77 (C-9′), 54.98 (C-OMe), 48.24 (C-8′), 46.58 (C-7′), 38.56 (C-8), 32.18 (C-7)
